# Phytochemical and Safety Evaluations of *Zingiber ottensii* Valeton Essential Oil in Zebrafish Embryos and Rats

**DOI:** 10.3390/toxics9050102

**Published:** 2021-05-03

**Authors:** Wisit Thitinarongwate, Raktham Mektrirat, Wutigri Nimlamool, Parirat Khonsung, Surachai Pikulkaew, Siriporn Okonogi, Puongtip Kunanusorn

**Affiliations:** 1 Department of Pharmacology, Faculty of Medicine, Chiang Mai University, Chiang Mai 50200, Thailand; wisit.vetcmu@gmail.com (W.T.); wutigri.nimlamool@cmu.ac.th (W.N.); wparirat@yahoo.com (P.K.); 2 Graduate School, Chiang Mai University, Chiang Mai 50200, Thailand; 3 Department of Veterinary Biosciences and Public Health, Faculty of Veterinary Medicine, Chiang Mai University, Chiang Mai 50100, Thailand; raktham.m@cmu.ac.th; 4 Research Center for Pharmaceutical Nanotechnology, Faculty of Pharmacy, Chiang Mai University, Chiang Mai 50200, Thailand; surapikulkaew@gmail.com; 5 Department of Food Animal Clinic, Faculty of Veterinary Medicine, Chiang Mai University, Chiang Mai 50100, Thailand; okng2000@gmail.com; 6 Department of Pharmaceutical Sciences, Faculty of Pharmacy, Chiang Mai University, Chiang Mai 50200, Thailand

**Keywords:** *Zingiber ottensii* Valeton, zebrafish, embryotoxicity, teratogenicity, rats, acute oral toxicity

## Abstract

*Zingiber ottensii* Valeton (ZO) exhibits pharmacological activity and has long been used in traditional medicine. However, reports about its safety profiles are limited. The present study aimed to evaluate the phytochemical profile and the toxic effects of ZO essential oil on the development of zebrafish and acute oral toxicity in rats. The essential oil was isolated from ZO rhizomes, and phytochemicals were analyzed using a gas chromatography–mass spectrometer (GC–MS). The embryotoxic and teratogenic effects of ZO essential oil were evaluated in zebrafish embryos and larvae and the acute oral toxicity was determined in rats. GC–MS results showed the essential oil contained zerumbone as a major phytoconstituent (24.73%). The zebrafish embryotoxicity of ZO essential oil appeared to be concentration- and time-dependent manner, with a moderate LC_50_ (1.003 µg/mL). Teratogenicity in zebrafish embryos also included morphological defects, decreased hatchability, and reduced heart rate. In rats, ZO essential oil (2000 mg/kg, p.o.) resulted in no mortality or significant toxicities. These findings suggest that ZO has embryotoxic and teratogenic effects in zebrafish embryos but does not result in death or acute oral toxicity in rats. Further long-term toxicity studies are needed to confirm the safety of products developed from ZO essential oil.

## 1. Introduction

Various plants have been shown to possess significant pharmacological activity and to provide many health benefits, both in preclinical studies (in vitro and in vivo models) and in clinical studies in humans [1,2,3]. Additionally, medicinal plants have been used as alternative treatments to cure illness in Ayurvedic and Thai traditional medicine. Presently, the use of medicinal plants is increasing as their natural compounds are believed to be safer than modern medicines. However, the toxicological profiles of most medicinal plants have not been completely determined. Many plant-derived treatments may result in harmful effects in humans, including carcinogenic, mutagenic, and teratogenic effects [4,5]. Toxicity testing in a diverse range of in vitro studies using animal models is crucial and includes experimental screening methods for determining the safety profile of medicinal plants. For example, acute oral toxicity test in rodents is widely used to evaluate acute toxicity of a drug or an herb. These tests can include evaluation of the LD_50_ of the tested substance. Although rodents, rabbits, and sheep have traditionally been used for studying toxicity in embryonic development. However, using these animals to study consumes time and expense. Thus, there is an effort to find an alternative animal model for the toxicity study of medicinal plants in embryonic development.

*Danio rerio*, commonly called zebrafish, is a small tropical freshwater fish in the Cyprinidae family. In recent times, animal models using zebrafish are becoming popular and reliable models widely used in biological researches such as in the fields of genetic development, transgenesis, and toxicology [6]. Zebrafish embryos are useful for evaluating vertebrate development because the developmental steps in the zebrafish embryo correspond to other higher vertebrates’ embryogenesis, including humans [7]. The egg of zebrafish was transparent, which allows the direct observation of developmental stages (from fertilization, embryogenesis, and organogenesis to larva hatching) and assessment of endpoint morphological changes in toxicity studies [8]. Moreover, animal models using zebrafish have many advantages including low husbandry cost, requiring small housing spaces, having a small number of chemical compounds and test drugs, short breeding cycle (5–7 days) and higher fecundity (with 200–300 eggs per one pair of adult fish), and are suitable for high throughput screening. These benefits of the zebrafish model are the reasons for the popularity of using these models as alternative models, in comparison to some vertebrate toxicity assessment models [9,10,11].

*Zingiber ottensii* (ZO) Valeton, locally famous as Plai Dam or Plai Muang (Bangkok) and Pu Loei Dum (northern Thailand), can be found in Southeast Asia, including Indonesia, Malaysia, and Thailand [12,13]. In Malay traditional medicine, midwives in Perak commonly make the poultice from leaves and rhizomes of ZO before applying it to the body of the confinement’s women for postpartum care. ZO leaves are also used as the poultice for lumbago [14]. In Thai traditional medicine, ZO rhizomes are used to treat gastrointestinal diseases (peptic ulcers and stomachache), constipation, myalgia, sprain, bruising/contusion, and wounds. Moreover, essential oil from ZO rhizomes has been used as a topical agent for Thai traditional massage. Although various pharmacological activities including antidiabetic, anticancer, and antimicrobial activities of ZO have been reported [15,16]. In addition, the major phytochemical components of essential oil of ZO rhizomes of Malaysia are reported to be terpene compounds [17]. However, the reports about phytochemical characteristics of essential oil of ZO rhizomes found in Thailand and its toxicological profiles including embryotoxicity, teratogenicity, and acute oral toxicity in animal models are still lacking. Thus, the present study aimed to identify the phytochemical profile of ZO essential oil from ZO grown in Thailand and evaluate its toxicity in in vivo models using both zebrafish embryos and rats. These findings of this study are intended to provide part of the data necessary to confirm the safety of new products developed using ZO essential oil in the future.

## 2. Materials and Methods

### 2.1. Plant Material

Rhizomes of *Zingiber ottensii* (ZO) Valeton were harvested at Saluang Subdistrict, Mae Rim District, Chiang Mai, Thailand (Lat 19°01′57.719″ N and Long 98°88′46.0119″ E) in March 2020, two years after planting. Plant authentication was carried out by the Faculty of Pharmacy, Chiang Mai University where the voucher specimen (000109) was deposited. The fresh rhizomes were washed, chopped into very small pieces, then kept in a storage box at room temperature before the extraction.

### 2.2. Distillation and Chemical Composition Analysis of ZO Essential Oil

One kg of ZO fresh rhizomes and 1 L of distilled water were used to obtain ZO essential oil by hydrodistillation extraction using a Clevenger apparatus over a period of 3 h. The ZO essential oil was then collected and stored in an airtight dark bottle at 4 °C until use. Gas chromatography–mass spectrometry (GC–MS) was used to identify the chemical components found in ZO essential oil. The system was comprised of a 7890A gas chromatograph (Agilent, Santa Clara, CA, USA) equipped with a 5975C mass-selective detector (Agilent, Santa Clara, CA, USA) using a DB5-MS column (30 m × 0.25 mm i.d. × 0.25 µm film thickness). The ionization energy was 70 eV and the ion source temperature was 230 °C. The oven temperature was initially set at 50 °C, then slowly raised to 220 °C over a period of 45 min (4 °C/min) with helium as the carrier gas. The flow rate of the carrier gas was set at 1 mL/min using a split mode (split ratio 500:1). The injection temperature was 250 °C, and the detector temperature was 280 °C. Identification of the ZO essential oil components was based on the comparison of their retention times and their mass spectra by matching with standard reference database and library, which included NIST Mass Spectral Database (2008) and W8N08 library (John Wiley & Sons, Inc., Hoboken, NJ, USA). Each component’s percentage was calculated based on the total area of all peaks obtained from the ZO essential oil.

### 2.3. Laboratory Animal Care and Maintenance

Wild-type zebrafish (*Danio rerio*) were purchased from a local ornamental fish shop, that normally supplies zebrafish used for studying in the aquatic laboratory of the Faculty of Veterinary Medicine, Chiang Mai University, Chiang Mai, Thailand. Maintenance of zebrafish was in accordance with the internationally accepted guideline (OECD 236) [18], with minor modifications. Adult zebrafish were maintained in an 80 L glass tank with a maximum density of 1 g fish/L filtered tap water (pH 6.9–7.4, 26 ± 1 °C) with a 14:10 h of the light–dark cycle in the Aquatic Medicine Room located of the Faculty of Veterinary Medicine, Chiang Mai University. The zebrafish were fed twice a day: frozen brine shrimps (*Artemia*) in the morning and commercial dry flake food in the afternoon. Water was changed and feces removed from each tank daily. Water parameters, including temperature, pH, nitrite, nitrate, and ammonia content, were also monitored daily. All zebrafish were quarantined for at least 3 months before using them in the experiments. They were selected and graded into many groups depending on health status and fertility. Only zebrafish with the best quality were selected to be used as the breeders. These zebrafish were acclimatized for 4 weeks before initial breeding. All experiments were approved by the Animal Ethics Committee of the Faculty of Veterinary Medicine, Chiang Mai University, Thailand (Permit No. R8/2563, 17 July 2020).

Female Sprague Dawley rats (*Rattus norvegicus*) (age 8–12 weeks/180–200 g) were purchased from Nomura Siam International Co. Ltd., Bangkok, Thailand. All the animals were kept in an animal room maintained under environmentally controlled conditions of 24 ± 1 °C, 50 ± 10% relative humidity, and a 12:12 h light–dark cycle. They had free access to drinking water and a standard pelleted diet and were acclimatized for at least one week before the start of the experiments. All experiments were approved by the Animal Ethics Committee of the Faculty of Medicine, Chiang Mai University, Thailand (Permit No. 22/2563, 9 July 2020).

### 2.4. Zebrafish Breeding and Embryo Care

The evening of the day before breeding the healthy and active adult male and female wild-type zebrafish (ratio 1:2) with high ability to produce fertilized eggs were selected for spawning and were moved to a spawning tank equipped with 5 L of filtered tap water and fitted with spawning enhancers consisting of marbles and a spawn trap. Mating occurred the next morning within 30–60 min after the lights were turned on. The zebrafish were then removed from the spawning tank and placed back into their resting glass tank. Spawning enhancers were removed from the spawning tank. Water was poured out slowly from one corner of the spawning tank, and the fertilized eggs were collected with a plastic pipette and transferred to clean Petri dishes containing embryo water prepared with minor modifications of the previously reported protocol [19]. The fertilized embryos were carefully washed with sea salt egg water (60 mg/L sea salt and 2 mg/L methylene blue) to remove debris [20], while unhealthy and dead embryos were removed by aspiration using a plastic pipette. Fertilized embryos were kept at 26.5 °C and allowed to develop for 6 h postfertilization (hpf). Microscopic observation was accomplished using a stereomicroscope (Nikon, Tokyo, Japan) to observe embryo development prior to treatment [21].

### 2.5. Zebrafish Embryonic Toxicity Test

#### 2.5.1. Dose–Response Embryotoxicity and Median Lethal Concentration (LC_50_)

Embryotoxicity in the zebrafish was assessed by measuring the mortality rate of the zebrafish embryos. Exposure of the zebrafish embryos to the extract was performed according to the method described in OECD 236 [18]. At 6 hpf, healthy zebrafish embryos were selected for subsequent experiments. Overall, 20 fertilized eggs for each concentration treatment were transferred to individual wells of 24-well plates. The embryos were exposed to various concentrations of ZO essential oil containing 0.1% dimethyl sulfoxide (DMSO) diluted in 2 mL of embryo water. It was serially diluted via twofold serial dilution to produce five different concentrations of ZO essential oil (3.91, 1.95, 0.98, 0.49, and 0.24 µg/mL). The control (untreated) group was exposed to 2 mL of embryo water containing only 0.1% DMSO. The experiment was duplicated, each performed with three independent replicates. The embryos were observed under a stereomicroscope every 24 h. Death of an embryo was indicated by coagulation and/or absence of a heartbeat. The number of dead embryos in each concentration at 96 hpf was recorded, and the mortality rate was calculated. The GraphPad Prism8 [log (inhibitor) vs. normalized response-variable slope nonlinear model] was used to calculate the 50% lethal concentration (LC_50_) values for ZO essential oil [22].

#### 2.5.2. Time–Kill Analysis

The time–kill kinetics of ZO essential oil were determined by analyzing the time–kill rates of the zebrafish embryos. Dead embryos were examined under a stereomicroscope, and the time–kill analysis was conducted at 24, 48, 72, 96, and 120 hpf. Survival rates and the median survival time were also evaluated [20].

### 2.6. Zebrafish Teratogenicity Test

#### 2.6.1. Evaluation of Morphological Characteristics

The teratogenicity of the zebrafish was evaluated both in embryos and in larvae after five days (120 hpf) exposure to five different concentrations of ZO essential oil by observing morphological changes and developmental abnormalities. After treatment, the embryos and larvae were examined every 24 h using a stereomicroscope. Embryonic and larval morphology was determined according to OECD Test Guideline 236 [18], and the normal development of the embryos and larvae was compared using the method of Kimmel et al. (1995) [7], as previously described. Examples of teratogenicity identified included deformities in somite otolith and eyes, failure of tail detachment, absence of heartbeat or blood circulation, yolk sac or pericardial edema, yolk sac malabsorption, and skeletal malformation and delayed hatching [23]. The malformed embryos and larvae were captured with an Olympus digital camera (OM-D E-M10 Mark III), and the teratogenicity was evaluated by the percentage of embryos or larvae with abnormalities to the number remaining of normal embryos, as previously described [20].

#### 2.6.2. Evaluation of Zebrafish Embryos Hatchability

The hatchability of the zebrafish embryos treated with different concentrations of ZO essential oil (0–3.91 µg/mL) was determined between 48 and 120 hpf using a stereomicroscope. The hatching success of embryos was determined by chorion rupture releasing the larvae into the embryo water. Hatchability rates were determined by comparing the number of hatched embryos with the total number of embryos tested.

#### 2.6.3. Evaluation of Zebrafish Larvae Heart Rates

The number of heartbeats of larvae between 48 and 120 hpf treatments with ZO essential oil (0–3.91 µg/mL) was determined in this experiment. At 72 hpf, the heartbeat count was conducted using a stereomicroscope connected to a computer and digital camera device for video recording. Counting was performed using a mechanical counter and stopwatch. The heart rate was expressed as beats per minute (bpm) [23,24].

### 2.7. Acute Oral Toxicity Study in Rats

The acute oral toxicity of ZO essential oil was evaluated in rats following internationally accepted guidelines (OECD Test Guideline 420) [25]. The female rats used in the experiment were randomly selected and marked on the tail for individual identification. A single high dose of 2000 mg/kg of ZO essential oil was administered by oral gavage to one rat following 12 h of fasting. 48 h later, the same dose was administered to another four rats, for a total of five treated rats. The negative control group of five rats was treated in parallel with the vehicle (0.9% saline). Food was provided to all rats approximately 1 h after administration.

All rats were observed in detail periodically and daily for 14 days for any toxic effects [26]. Intake of water and food, along with body weight, were measured daily [25]. Mortality, behavioral pattern, physical appearance changes, injuries, pain, and signs of illness were monitored daily during the period [27].

After 14 days, all the rats were sacrificed, and their vital organs, including heart, kidneys, liver, lung, and spleen were removed, weighed, and microscopically examined. All vital organs isolated from each rat were fixed in 10% buffered formalin before being subjected to further histopathological evaluation. The relative organ weight of each animal was calculated as follows:

Relative organ weight = organ weight (g)/body weight of the rat (g) × 100

### 2.8. Statistical Analysis

In zebrafish embryotoxicity and teratogenicity tests, the experiments were conducted with three independent replications. The mortality of zebrafish embryos and teratogenicity test results were compared by means of one-way analysis of variance (ANOVA) and Tukey’s multiple comparison test. Student’s *t*-test was used for comparisons between two experimental groups in the acute oral toxicity study using SPSS Statistical Package version 22 (IBM, Armonk, NY, USA). Data are presented as mean ± standard deviation (SD). Statistical significance of differences was set at *p* < 0.05. Differences between the five groups in rates of time to outcome were compared using the Kaplan–Meier curve, and the log-rank test for significance and statistical analysis of LC_50_ was calculated using GraphPad Prism8 Software (San Diego, CA, USA).

## 3. Results

### 3.1. Chemical Compositions of the Zingiber ottensii (ZO) Valeton Essential Oil

**Figure 1:**
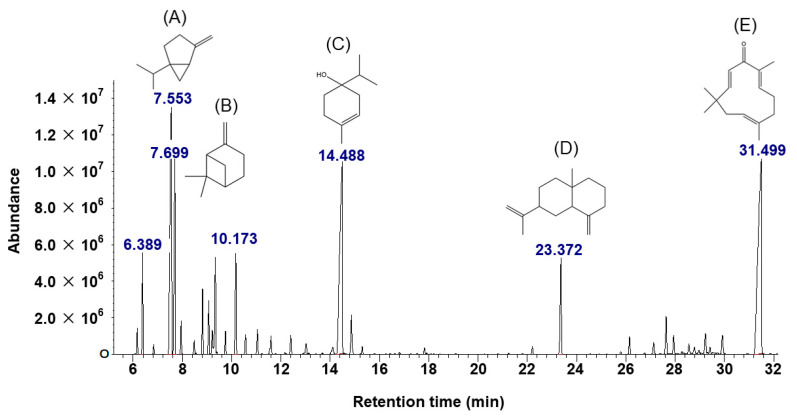
GC–MS chromatogram of ZO essential oil. Phytochemicals were identified by the GC–MS method. Chemical structures of the five major compounds from ZO essential oil included (**A**) sabinene, (**B**) β-pinene, (**C**) terpinen-4-ol, (**D**) β-selinene, and (**E**) zerumbone.

The rhizomes of ZO were subjected to hydrodistillation and yielded 0.24% (*w*/*w*) of essential oil with a pale yellowish color and camphoraceous odor. Phytochemicals were characterized using the GC–MS method. The peak numbers were recorded according to the retention time and percentage of each compound (Table 1). Most constituents were terpenoids, which consisted mainly of 21 monocyclic monoterpenoids and seven sesquiterpenes. Chromatograms showed major components’ identifiable spectra (Figure 1). The compound present in the highest quantity was zerumbone (24.73%), followed by terpinen-4-ol (18.75%), sabinene (15.19%), and β-pinene (7.95%).

### 3.2. Dose–Response Embryotoxicity in Zebrafish and LC_50_

**Figure 2:**
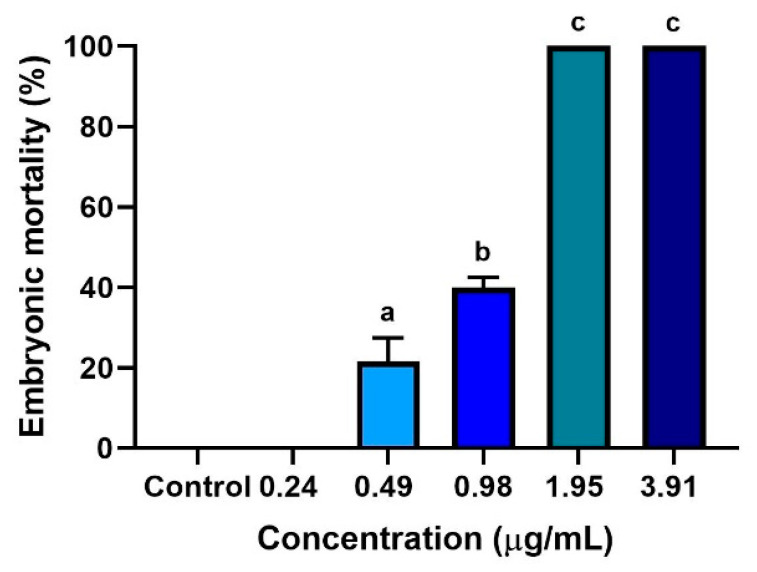
The embryotoxicity of different concentrations of ZO essential oil in embryonic zebrafish. Zebrafish embryos were treated with a series of twofold dilution concentrations (0.24–3.91 µg/mL). Percentages of embryonic mortality were calculated from embryo deaths after exposure to ZO essential oil at 0.24, 0.49, 0.98,1.95, 3.91 µg/mL, and 0.1% DMSO (control). Data represent the mean ± SD of three independent experiments (*n* = 60 embryos/group). Experiments were analyzed using one-way ANOVA and Tukey’s multiple comparison test. Different lowercase letters indicate significant differences between groups (*p* < 0.05).

Embryotoxicity was evaluated at five different concentrations of ZO essential oil (3.91, 1.95, 0.98, 0.49, and 0.24 µg/mL). Coagulation and the absence of heartbeat in zebrafish embryos were indicative of mortality [4]. The results showed that the toxic effect of ZO essential oil appeared to occur in a concentration-dependent manner. The mean mortality of zebrafish at 96 hpf is shown in Figure 2. The lowest dose of ZO essential oil (0.24 µg/mL) and 0.1% DMSO (control) caused no mortality in the zebrafish embryos. By contrast, a significantly increased mortality rate (*p* < 0.05) was observed in zebrafish embryos exposed to 0.49, 0.98, 1.95, and 3.91 µg/mL of ZO essential oil, when compared with the lowest concentration of ZO essential oil (0.24 µg/mL). No viable zebrafish embryos were observed in the groups treated with 1.95 and 3.91 µg/mL ZO essential oil. The LC_50_ value of ZO essential oil was 1.003 µg/mL.

### 3.3. Time–Kill Analysis in Zebrafish Embryos

**Figure 3:**
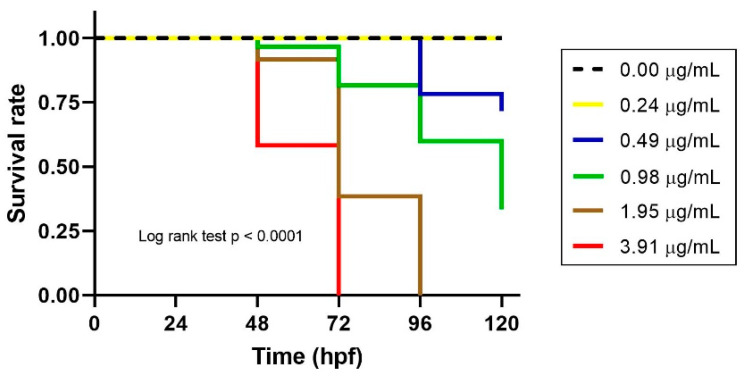
The embryotoxicity of different concentrations of ZO essential oil in the time–kill analysis of embryonic zebrafish. Zebrafish embryos were treated with 0.1% DMSO (control), and 0.24, 0.49, 0.98, 1.95 and 3.91 µg/mL of ZO essential oil. The Kaplan–Meier curve shows the average survival rate in six different groups in the three independent experiments (*n* = 60 embryos/group). The log-rank test was used for statistical analysis (*p* < 0.0001).

Time–kill analysis of five different concentrations of ZO essential oil in a series of twofold dilution concentrations over the range of 0.24–3.91 µg/mL was conducted to evaluate the kinetic killing in zebrafish embryos over the course of 24–120 hpf. The Kaplan–Meier curve was used to display the relationship between time (hpf) to zebrafish embryo death (Figure 3). The results showed that the killing potency of ZO essential oil appeared to occur in a time-dependent manner. The survival rate of zebrafish embryos treated with the lowest dose of ZO essential oil or with 0.1% DMSO (control) was 1.00. On the other hand, the survival rates of zebrafish embryos treated with 0.49 and 0.98 µg/mL of ZO essential oil were reduced to 0.72 and 0.33 at 120 hpf, respectively. Interestingly, the survival rates of zebrafish embryos treated with 3.91 and 1.95 µg/mL were decreased to zero at 72 and 96 hpf, respectively (log-rank test, *p* < 0.0001). The mean survival times of zebrafish embryos treated with 3.91 and 1.95 µg/mL of ZO essential oil were equal (72 hpf), while the mean survival time in the group treated with 0.98 µg/mL of ZO essential oil was 120 hpf.

### 3.4. Morphological Defects of Zebrafish Embryos

**Figure 4:**
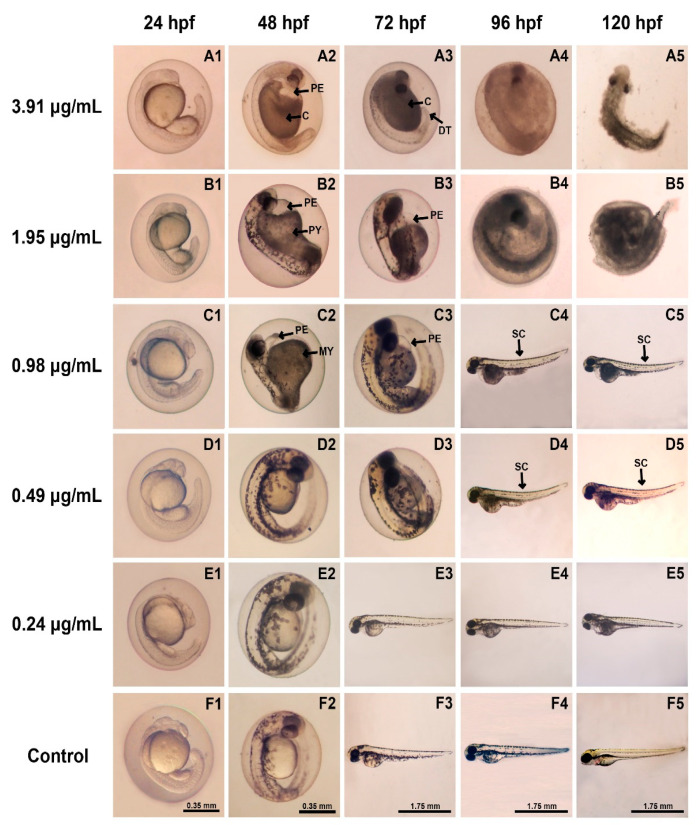
Morphological characteristics of zebrafish embryos and larvae treated with different concentrations of ZO essential oil or vehicle at different time points (24–120 hpf). Normal morphology was found in the group treated with the lowest concentrations and with vehicle (**E**,**F**). Typical malformations of zebrafish development were observed in embryos that had received ZO essential oil at a dose of 0.49–3.91 µg/mL (**A**–**D**). Abbreviations: PE, pericardial sac edema; C, coagulation; DT, dented tail; PY, poor reabsorption of yolk sac; MY, malformation of yolk sac; SC, spinal curvature.

Determination of teratogenicity was performed using five different concentrations of ZO essential oil (3.91, 1.95, 0.98, 0.49, and 0.24 µg/mL) and 0.1% DMSO (control) at various time points over the period 24–120 hpf (Figure 4). The rate of teratogenic malformation resulting from ZO essential oil in zebrafish embryos is presented in Table 2. The results showed no teratogenic abnormalities in the control group of zebrafish embryos or in the group treated with 0.24 µg/mL of ZO essential oil (Figure 4E,F). However, all embryos and larvae were found to express morphological abnormalities when 3.91, 1.95, and 0.98 µg/mL of ZO essential oil were present at 48, 72, and 96 hpf, respectively (Table 2). Notably, zebrafish embryo’s accumulative abnormalities were found in the groups treated with 0.49–3.91 µg/mL of ZO essential oil. Morphological defects included pericardial sac edema, coagulation, dented tail, poor reabsorption of the yolk sac, malformation of the yolk sac, and spinal curvature (Figure 4A–D).

### 3.5. Hatchability of Zebrafish Embryos

**Figure 5:**
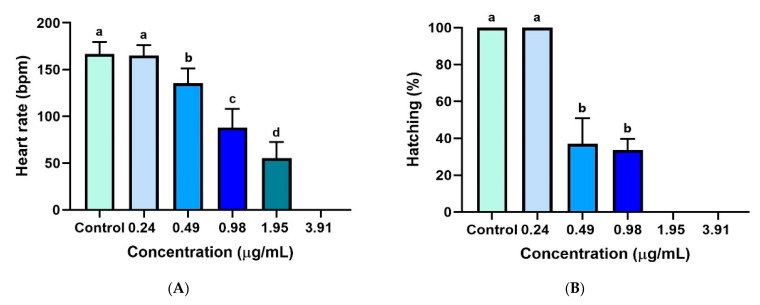
Assessment of hatchability and heart rate of zebrafish embryos. (**A**) Percentage of embryos hatching of zebrafish treated with ZO essential oil. (**B**) Correlation between the dose of ZO essential oil and zebrafish larvae heart rate (bpm) at 72 hpf. Data are presented as mean ± SD of three independent experiments (*n* = 60 embryos/group). Data were analyzed using one-way ANOVA and Tukey’s multiple comparison test. Different lowercase letters indicate significant differences between groups (*p* < 0.05).

The hatching of zebrafish embryos indicates successful embryonic development of the embryos after 48 hpf [28]. The hatching rates of zebrafish embryos treated with five different concentrations of ZO essential oil (3.91, 1.95, 0.98, 0.49, and 0.24 µg/mL) and 0.1% DMSO (control) are presented in Figure 5A. As the concentration increased, the hatching rate of treated embryos became lower. No hatching of any zebrafish embryos was observed in the 3.91 and 1.95 µg/mL treated groups. All of the zebrafish embryos treated with 0.24 µg/mL of the ZO essential oil or vehicle were successfully hatched.

### 3.6. Heart Rates of Zebrafish Embryos

The normal heart rate of zebrafish embryos ranges from 120 to 180 bpm [29,30]. The zebrafish heart rates at 72 hpf with different concentrations are shown in Figure 5B. There was no significant difference between the means of the heart rates of the ZO essential oil (0.24 µg/mL)-treated group and the 0.1% DMSO-treated group (control). The average heart rate declined at higher concentrations. No heartbeat was detected at 72 hpf in any zebrafish embryos treated with 3.91 µg/mL of ZO essential oil due to embryo death.

### 3.7. Lethality and Behavioral Analysis of Rats

Lethality assessment of rats treated with 2000 mg/kg of ZO essential oil revealed that no death of any animal occurred over 14 days. Some changes of general appearance and behavior seemed to be found at 6 h and 12 h after ZO essential oil administration, including sedation, lethargy, and ataxia. However, all the observed behavioral changes were later recovered to normal (Table 3).

### 3.8. Body Weight, Food and Water Consumption, and Relative Organ Weight Analysis

The rats’ final weight, the percentage of body weight gain, and food and water intake of the control and treatment groups were monitored and calculated (Table 4). There were no significant differences observed between the control and treatment groups. Additionally, the relative organ weights of all organs examined were similar among the groups (Table 5).

### 3.9. Macroscopic and Histopathological Analysis

**Figure 6:**
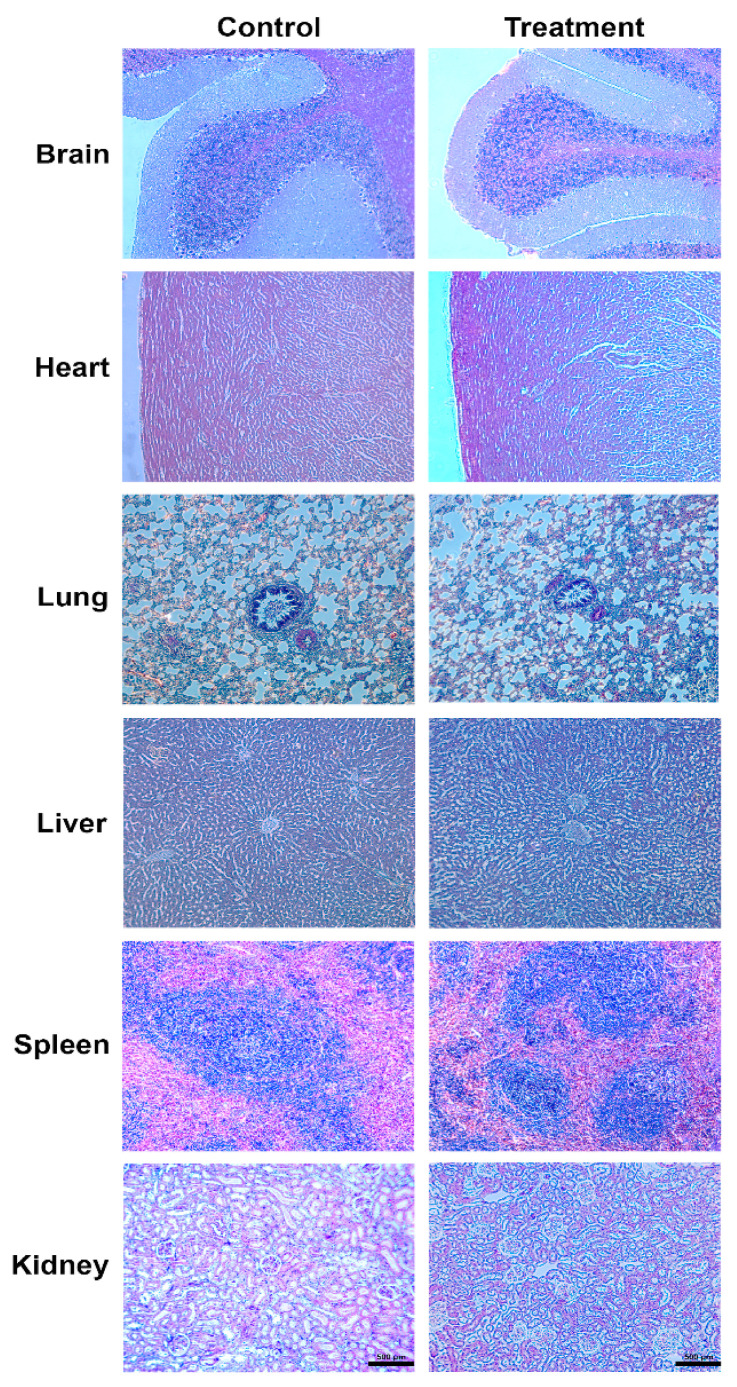
Histopathological examination of vital organs of rat treated with 2000 mg/kg of ZO essential oil, compared to the control group in acute oral toxicity study (H&E; ×100).

Macroscopic evaluation of vital organs of rats treated with 2000 mg/kg of ZO essential oil found no characteristic changes that were different from the control group. The histopathological analysis of vital organs, including the brain, heart, lung, liver, spleen, and kidney, revealed no significant structural differences between the treatment and control groups (Figure 6).

## 4. Discussion

GC–MS analysis revealed that the main components of *Zingiber ottensii* (ZO) Valeton essential oil were sesquiterpenes of which zerumbone was the major compound (24.73%), followed by monoterpenes including terpinene-4-ol (18.75%), sabinene (15.19%), and β-Pinene (7.95%) (Table 1). These findings are in line with the phytochemical compositions of ZO cultivated in Johor, Malaysia, and Phetchaburi Province, Thailand, where major constituents have been reported to be zerumbone (25.63% and 40.14%), terpinene-4-ol (16.81% and 11.17%), sabinene (7.20% and 6.48%), and β-Pinene (5.08% and 4.32%) [17,31]. The most abundant compound of ZO cultivated in Bandung, Indonesia, however, is terpinene-4-ol (16.55%), followed by zerumbone (14.23%) with some other phytochemical components including longifolenaldehyde (1.33%) and 2,5,9-trimethyl-cycloundeca-4,8-dienone (1.00%) [32]. The differences in the amounts and phytoconstituents of the essential oil may be due to differences in cultivation areas, growing seasons, harvest times, and environmental conditions [33]. Interestingly, zerumbone and terpinene-4-ol have been reported to possess a broad spectrum of beneficial biological effects, e.g., anti-inflammatory and antioxidant effects [34,35]. Those effects make it attractive to develop pharmaceutical products from ZO essential oil, which can could potentially increase the value of the herbs as well as local farmers’ income. However, an important initial step in the development of any drug or herbal product is to confirm its safety, generally by in vivo toxicity studies. In particular, there is still a lack of scientific evidence demonstrating the safety profile of ZO essential oil in terms of embryonic and teratogenic toxicity and acute oral toxicity. The zebrafish model can also be used in high throughput and valid screening models to study the embryotoxic and teratogenic effects. Such studies require only small amounts of test substances, short duration of study, are low cost and are not so complicated to perform, compared with toxicity models in rats. On the other hand, models to study the development toxicity in rats require higher amounts of test substances, longer duration of study, are higher cost and are very complicated to perform. Due to these reasons, zebrafish models were used, instead of models to study the development toxicity in rats, as the screening models to study the embryotoxic and teratogenic effects of ZO essential oil. The acute oral toxicity in rats is also another well-known model. It is used to provide information on health hazards likely to arise from short-term exposure by the oral route of any test substance and is a step in establishing a dosage regimen in subchronic and other toxicity studies.

Prior to performing safety studies in a rodent model, a toxicological assay of vertebrate zebrafish has been proposed as a tractable model for screening potentially suitable phytochemical concentrations in whole more complex organisms. The present study provides a toxicity assessment of ZO essential oil in zebrafish embryos. This study observed no embryotoxic mortality or malformation when using the lowest concentration of ZO essential oil (0.24 µg/mL), indicating that this concentration is not toxic to fish embryos. In contrast, increases in ZO essential oil concentrations were found to be related to higher rates of embryonic mortality and death of all embryos at the concentrations of 3.91 and 1.95 µg/mL, at 96 and 72 hpf, respectively (Figure 2). Notably, higher doses of ZO essential oil were observed to have an increase in embryotoxicity over the course of ZO treatment. These results indicate that in zebrafish embryos the toxicity of ZO essential oil appears to be concentration- and time-dependent manner. These results are in line with studies of other plants in the Zingiberaceae family, including the essential oil of *Zingiber cassumunar* Roxb. and the methanolic extract of *Curcuma longa* Linn., in which embryotoxicity of zebrafish embryo was also observed to be concentration- and time-dependent manners [20,23]. Additionally, since zerumbone was found to be a major compound of ZO essential oil in this study, it could be responsible for the embryotoxic and teratogenic effects on zebrafish. Moreover, sabinene and terpinen4-ol found in ZO essential oil could also be responsible for these effects since they were also found to be the major compounds of cassumunar ginger oil that also possessed embryotoxic and teratogenic effects [20].

Development deformities in zebrafish embryos were observed in the groups treated with 0.49–3.91 µg/mL of ZO essential oil. Deformities of body parts or organs included a dented tail, failure of tail detachment, spinal curvature, pericardial sac edema, and poor reabsorption of yolk (Figure 4). The abnormalities increased noticeably with prolonged exposure to ZO essential oil for 24–120 hpf (Table 2). These findings suggest that the accessibility of the ZO essential oil introduced into the animal’s body increases with the duration of exposure and eventually leads to embryotoxicity. This phenomenon may be caused by the alternation of the embryo protective layer (chorion) protein, which may result in opening and widening of the chorion pore channel during normal development of the zebrafish leading to weakened and damaged chorion, which, in turn, allows more ZO essential oil to pass into the embryos [23,36]. Furthermore, terpenoid found in ZO essential oil is a compound with nonpolar properties and a low molecular weight, making it easier for it to pass through the cytoplasmic membrane of zebrafish embryos [20].

Edema formation may be caused by overhydration of the zebrafish embryo due to osmoregulatory system problems related to toxicant accumulation [37]. Cardiac edema was observed in this study (Figure 4(A2,B2,B3,C2,C3)), and most embryos exhibited cardiovascular abnormalities, including a reduction of heart rate (Figure 5B) similar to previous reports [38].

A decrease in the hatching rate of zebrafish embryos and a delayed hatching process were observed, which are events correlated with increasing concentrations of ZO essential oil (Figure 5A). A low hatchability rate and delayed hatching process normally indicate growth retardation, which may be due to abnormal embryo development. In particular, it may be related to a decreased chorion breaking ability resulting from the inhibition of the tetraspanin gene (*cd63*), which causes a lack of secreted proteolytic enzymes necessary for chorion softening [39,40]. Obvious morphological abnormalities could be observed in these larvae [41].

Additionally, as an initial step in drug development, we performed an acute oral toxicity study in rats to evaluate the safe dose of this medicinal plant in an in vivo model, which provides for more toxic expression, including general appearance and behavioral changes, than in vitro assay. Specifically, in our current study, each rat received a single dose of ZO essential oil (2000 mg/kg), and the results showed no mortality of any rats, no significant changes in the percentage of body weight gain, daily food, and water consumption, and no differences in relative organ weight along with normal organ appearance and structures in gross pathology and histopathology analysis, when compared to the control group (Table 3, Table 4 and Table 5). However, alterations in some signs of general appearance (sedation, lethargy, and ataxia) seemed to be found (Table 3), but all these signs disappeared, and the animals returned to normal 12–24 h post administration. These temporary effects could imply that ZO essential oil may possess CNS-depressant or sedative-hypnotic effects. This result is in agreement with a previous in vivo study of terpenes, e.g., terpinen-4-ol, which is a compound that has sedative and anesthetic effects in fish [36]. Furthermore, the inhalation of sabinene and 1,8 cineole has been shown to exhibit strong sedative activity and to reduce locomotor activity in mice [42]. Oral administration of α- and β-pinene have also been demonstrated to induce mild sedation [43].

The present study revealed the monoterpene and sesquiterpene compounds in ZO essential oil and determined the toxicological profiles of the essential oil in zebrafish embryos and larvae. However, in the acute oral toxicity study in rats, ZO essential oil exhibited only temporary alteration of some general appearance characteristics without other significant toxicity or inducing death. Since routes of administration of ZO essential oil in the zebrafish model (topical route) and the rat model (oral route) were different, the toxic concentrations of ZO essential oil in the zebrafish model cannot be directly compared with the doses of ZO essential oil in the rat model. Moreover, the teratogenic effect of any substance depends on the ability of that substance to cross the placental barrier. In addition, some compounds found to be toxic in a rat embryotoxicity study were also found to cause deformities in zebrafish [44]. For these reasons, the use of ZO essential oil should be concerned in pregnant women. However, further confirmation of safety profiles of ZO essential oil in long-term toxicity studies, especially with other higher-order vertebrates, is needed. Such toxicological studies using different animal species are necessary to confirm the safety of any novel commercial pharmaceutical products containing ZO essential oil.

## 5. Conclusions

The essential oil of *Zingiber ottensii* (ZO) Valeton contains terpene compounds, of which zerumbone is a major constituent. The embryotoxicity of ZO essential oil in zebrafish appears to be in a concentration- and time-dependent manner and to have a moderate LC_50_ (1.003 µg/kg). Teratogenic effects of ZO essential oil on zebrafish embryos include morphological defects, reduced hatchability, and decreased heart rate. In the acute oral toxicity study in rats, temporary changes in general conditions including sedation, lethargy, and ataxia seem to be found, but percent body weight gain, water and food consumption, and organ characteristics were unchanged, and there were no deaths of any rats. These preclinical study results provide crucial support for the safety profile of ZO essential oil, an initial step in drug and natural pharmaceutical development. However, further long-term toxicity studies are needed to confirm the safety of any newly developed products containing ZO essential oil.

## Figures and Tables

**Table 1:** Chemical composition of essential oil from *Zingiber ottensii* (ZO) Valeton identified by GC–MS analysis. The ZO essential oil was extracted from the rhizomes of ZO by simultaneous steam distillation and analyzed by GC–MS. RT: Retention time; MW: molecular weight.

Peaks	RT (Min)	Component	Formula	MW (g/moL)	Amount (%)
1	6.39	α-pinene	C_10_H_16_	136.23	2.94
2	7.55	sabinene	C_10_H_16_	136.23	15.19
3	7.69	β-pinene	C_10_H_16_	136.23	7.95
4	9.34	1,8-cineole	C_10_H_18_O	154.25	3.16
5	10.18	γ-terpinene	C_10_H_16_	136.23	3.73
6	14.49	terpinen-4-ol	C_10_H_18_O	154.25	18.75
7	14.87	α-terpineol	C_10_H_18_O	154.25	1.67
8	23.37	β-selinene	C_15_H_24_	204.35	4.48
9	29.24	α-eudesmol	C_15_H_22_O	222.37	0.95
10	31.50	zerumbone	C_15_H_22_O	218.33	24.73

**Table 2:** Teratogenic effect of ZO essential oil on zebrafish embryos at 24, 48,72, 96, and 120 hpf, which had received ZO essential oil at dose of 0.24, 0.49, 0.98, 1.95, and 3.91 µg/mL, and 0.1% DMSO (control) at 24, 48, 72, 96, and 120 hpf. Descriptive data show the percentages (mean ± SD) of teratogenic zebrafish embryos from three independent experiments (*n* = 60 embryos/group). ED = embryonic death.

Concentrations (µg/mL)	Hours Post Fertilization (hpf)				
	24	48	72	96	120
0	0	0	0	0	0
0.24	0	0	0	0	0
0.49	0	0	40.17 ± 2.25	75.36 ± 4.84	79.28 ± 8.24
0.98	0	18.99 ± 4.15	91.50 ± 8.10	100	100
1.95	0	47.30 ± 21.75	100	ED	ED
3.91	0	100	ED	ED	ED

**Table 3:** General appearance and behavioral observations of the control group (0.9% saline) and the treatment group (2000 mg/kg of ZO essential oil) over the period from 6 h to 14 days after administration. N = normal, + = detected and - = not detected.

Observation	Control Group (0.9% Saline)					Treatment Group (2000 mg/kg of ZO Essential Oil)				
	6 h	12 h	24 h	7 d	14 d	6 h	12 h	24 h	7 d	14 d
Behavioral patterns	N	N	N	N	N	N	N	N	N	N
Skin and Fur	N	N	N	N	N	N	N	N	N	N
Eye and Ears	N	N	N	N	N	N	N	N	N	N
Mucous membrane	N	N	N	N	N	N	N	N	N	N
Heartbeat	N	N	N	N	N	N	N	N	N	N
Breathing	N	N	N	N	N	N	N	N	N	N
Sedation	-	-	-	-	-	+/-	+/-	-	-	-
Lethargy	-	-	-	-	-	+/-	+/-	-	-	-
Ataxia	-	-	-	-	-	+/-	+/-	-	-	-
Salivation	-	-	-	-	-	-	-	-	-	-
Diarrhea	-	-	-	-	-	-	-	-	-	-
Convulsion	-	-	-	-	-	-	-	-	-	-

**Table 4:** Body weight and food and water intake of the control group (0.9% saline) and the treatment group (2000 mg/kg of ZO essential oil) during the 14 day observation period. Data shown are the mean ± SD (*n* = 5 rats/group). All data were analyzed using Student’s *t*-test (*p* < 0.05).

Parameters	Control Group (0.9% Saline)	Treatment Group (2000 mg/kg of ZO Essential Oil)
Initial weight (g)	185.00 ± 5.00	183.00 ± 4.47
Final weight (g)	213.60 ± 7.40	214.40 ± 3.43
Body weight gain (%)	12.62 ± 3.51	12.14 ± 1.67
Food intake (g/day)	13.89 ± 0.04	13.57 ± 0.73
Water intake (mL/day)	29.68 ± 2.27	31.85 ± 2.66

**Table 5:** Relative organ weights of the control group (0.9% saline) and the treatment group (2000 mg/kg of ZO essential oil). Data shown are the mean ± SD (*n* = 5 rats/group). All data were analyzed using Student’s *t*-test (*p* < 0.05).

Organ (g/100 g Body Weight)	Control Group (0.9% Saline)	Treatment Group (2000 mg/kg of ZO Essential Oil)
Brain	0.93 ± 0.09	0.89 ± 0.02
Heart	0.32 ± 0.01	0.30 ± 0.02
Liver	4.63 ± 0.22	4.51 ± 0.10
Kidney	0.50 ± 0.02	0.49 ± 0.01
Spleen	0.25 ± 0.02	0.25 ± 0.03
Thymus gland	0.21 ± 0.03	0.20 ± 0.04
Ovary	0.03 ± 0.01	0.03 ± 0.01
Uterus	0.25 ± 0.02	0.28 ± 0.06
Adrenal gland	0.02 ± 0.00	0.02 ± 0.00
Lung	0.52 ± 0.04	0.75 ± 0.23

## References

[1] Ruttanapattanakul J., Wikan N., Okonogi S., Na Takuathung M., Buacheen P., Pitchakarn P., Potikanond S., Nimlamool W. (2021). *Boesenbergia rotunda* extract accelerates human keratinocyte proliferation through activating ERK1/2 and PI3K/Akt kinases. Biomed. Pharmacother..

[2] Kunanusorn P., Teekachunhatean S., Sangdee C., Panthong A. (2009). Antinociceptive and anti-inflammatory activities of a chinese herbal recipe (DJW) in animal models. Int. J. Appl. Res. Nat. Prod..

[3] Arpornchayanon W., Gomonchareonsiri S., Chansakaow S., Wongpakaran T., Varnado P., Wongpakaran N. (2019). Acute effects of essential oil blend containing phlai oil on mood among healthy male volunteers: Randomized controlled trial. J. Complement. Integr. Med..

[4] Romagosa C.M.R., David E.S., Dulay R.M.R. (2016). Embryo-toxic and teratogenic effects of *Tinospora cordifolia* leaves and bark extracts in Zebrafish (*Danio rerio*) embryos. Asian J. Plant Sci. Res..

[5] Ng’uni T., Klaasen J.A., Fielding B.C. (2018). Acute toxicity studies of the South African medicinal plant *Galenia africana*. Toxicol. Rep..

[6] Lele Z., Krone P.H. (1996). The zebrafish as a model system in developmental, toxicological and transgenic research. Biotechnol. Adv..

[7] Kimmel C.B., Ballard W.W., Kimmel S.R., Ullmann B., Schilling T.F. (1995). Stages of embryonic development of the zebrafish. Dev. Dyn..

[8] Jayasinghe C.D., Jayawardena U.A. (2019). Toxicity assessment of herbal medicine using zebrafish embryos: A systematic review. Evid. Based Complement. Alternat. Med..

[9] Lammer E., Carr G., Wendler K., Rawlings J., Belanger S., Braunbeck T. (2009). Is the fish embryo toxicity test (FET) with the zebrafish (*Danio rerio*) a potential alternative for the fish acute toxicity test?. Comp. Biochem. Physiol. C Toxicol. Pharmacol..

[10] Mendis J.C., Tennakoon T.K., Jayasinghe C.D. (2018). Zebrafish embryo toxicity of a binary mixture of pyrethroid insecticides: D-tetramethrin and cyphenothrin. J. Toxicol..

[11] Halili J.F., Quilang J. (2011). The zebrafish embryo toxicity and teratogenicity assay. Philipp. Biota..

[12] Quattrocchi U. (2012). *Zingiber ottensii* Valeton. CRC World Dictionary of Medicinal and Poisonous Plants: Common Names, Scientific Names, Eponyms, Synonyms, and Etymology.

[13] Jansen P.C.M., de Guzman C.C.S.J. (1999). *Zingiber ottensii* Valeton. Plant Resources of South-East Asia No. 13: Spices.

[14] Burkill I.H., Birtwistle W., Foxworthy F.W., Scrivenor J.B., Watson J.G. (1935). A Dictionary of the Economic Products of the Malay Peninsula.

[15] Tiengburanatam N., Boonmee A., Sangvanich P., Karnchanatat A. (2010). A novel α-glucosidase inhibitor protein from the rhizomes of *Zingiber ottensii* Valeton. Appl. Biochem. Biotechnol..

[16] Karnchanatat A., Tiengburanatam N., Boonmee A., Puthong S., Sangvanich P. (2011). Zingipain, a cysteine protease from *Zingiber ottensii* Valeton rhizomes with antiproliferative activities against fungi and human malignant cell lines. Prep. Biochem. Biotechnol..

[17] Sirat H.M., Nordin A.B. (1994). Essential Oil of *Zingiber ottensii* Valeton. J. Essent. Oil Res..

[18] OECD (2013). Guidelines for the Testing of Chemicals. Section 2-Effects on Biotic System Test (No 236 Fish Embryo Acute Toxicity (FET) Test).

[19] Zebrafish Embryo Medium. http://cshprotocols.cshlp.org/content/2011/8/pdb.rec12478.full.

[20] Mektrirat R., Yano T., Okonogi S., Katip W., Pikulkaew S. (2020). Phytochemical and safety evaluations of volatile terpenoids from *Zingiber cassumunar* Roxb. on mature carp peripheral blood mononuclear cells and embryonic zebrafish. Molecules.

[21] Pamanji R., Yashwanth B., Bethu M.S., Leelavathi S., Ravinder K., Rao J.V. (2015). Toxicity effects of profenofos on embryonic and larval development of Zebrafish (*Danio rerio*). Environ. Toxicol. Pharmacol..

[22] Gao X.-P., Feng F., Zhang X.-Q., Liu X.-X., Wang Y.-B., She J.-X., He Z.-H., He M.-F. (2014). Toxicity assessment of 7 anticancer compounds in zebrafish. Int. J. Toxicol..

[23] Alafiatayo A., Lai K.-S., Syahida A., Mahmood M., Shaharuddin N. (2019). Phytochemical evaluation, embryotoxicity, and teratogenic effects of *Curcuma longa* extract on zebrafish (*Danio rerio*). Evid. Based Complement. Alternat. Med..

[24] Lu S., Hu M., Wang Z., Liu H., Kou Y., Lyu Z., Tian J. (2020). Generation and application of the zebrafish heg1 mutant as a cardiovascular disease model. Biomolecules.

[25] OECD (2002). Guidelines for the Testing of Chemicals. Section 4-Health Effects (No 420 Acute Oral Toxicity—Fixed Dose Procedure).

[26] Balin P., Zanatta F., Jorge B., Leitão M., Kassuya R., Cardoso C., Kassuya C., Arena A. (2018). Toxicological evaluation and anti-inflammatory potential of an ethanolic extract from *Bromelia balansae* (Bromeliaceae) fruit. J. Ethnopharmacol..

[27] Syahmi A.R.M., Vijayarathna S., Sasidharan S., Latha L.Y., Kwan Y.P., Lau Y.L., Shin L.N., Chen Y. (2010). Acute oral toxicity and brine shrimp lethality of *Elaeis guineensis* Jacq., (oil palm leaf) methanol extract. Molecules.

[28] Lieschke G.J., Currie P.D. (2007). Animal models of human disease: Zebrafish swim into view. Nat. Rev. Genet..

[29] De Luca E., Zaccaria G.M., Hadhoud M., Rizzo G., Ponzini R., Morbiducci U., Santoro M.M. (2014). ZebraBeat: A flexible platform for the analysis of the cardiac rate in zebrafish embryos. Sci. Rep..

[30] Kumar R.B.S., Kar B., Dolai N., Haldar P. (2013). Study on developmental toxicity and behavioral safety of *Streblus asper* Lour. bark on zebrafish embryos. Indian J. Nat. Prod. Resour..

[31] Thubthimthed S., Limsiriwong P., Rerk-am U., Suntorntanasat T. (2005). Chemical composition and cytotoxic activity of the essential oil of *Zingiber ottensii*. Acta Horti..

[32] Marliani L.S.A., Moelyono M.W., Halimah E., Pratiwi F.W., Suhardiman A. (2018). Essential oil components of leaves and rhizome of *Zingiber ottensii* Val. from Bandung, Indonesia. Res. J. Chem. Environ..

[33] Manochai B., Paisooksantivatana Y., Choi H., Hong J.H. (2010). Variation in DPPH scavenging activity and major volatile oil components of cassumunar ginger, *Zingiber montanum* (Koenig), in response to water deficit and light intensity. Sci. Horti..

[34] Kalantari K., Moniri M., Boroumand Moghaddam A., Abdul Rahim R., Bin Ariff A., Izadiyan Z., Mohamad R. (2017). A review of the biomedical applications of zerumbone and the techniques for its extraction from ginger rhizomes. Molecules.

[35] de Freitas Souza C., Baldissera M., Silva L., Geihs M., Baldisserotto B. (2018). Is monoterpene terpinen-4-ol the compound responsible for the anesthetic and antioxidant activity of *Melaleuca alternifolia* essential oil (tea tree oil) in silver catfish?. Aquaculture.

[36] Ali M., Saba S., Taite D., Emadi S., Irving R. (2017). The protective layer of zebrafish embryo changes continuously with advancing ages of embryo development(AGED). J. Toxicol. Pharmacolo..

[37] Cook L.W., Paradise C.J., Lom B. (2005). The pesticide malathion reduces survival and growth in developing zebrafish. Environ. Toxicol. Chem..

[38] Willaert A., Khatri S., Callewaert B., Coucke P., Crosby S., Lee J., Davis E., Shiva S., Tsang M., De Paepe A. (2011). GLUT10 is required for the development of the cardiovascular system and the notochord and connects mitochondrial function to TGF signaling. Hum. Mol. Genet..

[39] Murugesu S., Khatib A., Ahmed Q.U., Ibrahim Z., Uzir B.F., Benchoula K., Yusoff N.I.N., Perumal V., Alajmi M.F., Salamah S. (2019). Toxicity study on *Clinacanthus nutans* leaf hexane fraction using *Danio rerio* embryos. Toxicol. Rep..

[40] Trikić M.Z., Monk P., Roehl H., Partridge L.J. (2011). Regulation of zebrafish hatching by tetraspanin cd63. PLoS ONE.

[41] Braunbeck T., Böttcher M., Hollert H., Kosmehl T., Lammer E., Leist E., Rudolf M., Seitz N. (2005). Towards an alternative for the acute fish LC50 test in chemical assessment: The fish embryo toxicity test goes multi-species—An update. Altex.

[42] Dougnon G., Ito M. (2019). Sedative effects of the essential oil from the leaves of *Lantana camara* occurring in the Republic of Benin via inhalation in mice. J. Nat. Med..

[43] Felipe C., Albuquerque A., Pontes J., Melo J., Rodrigues T., Sousa A., Monteiro Á., Ribeiro A., Lopes J., Almeida R. (2018). Comparative study of alpha- and beta-pinene effect on PTZ-induced convulsions in mice. Fundam. Clin. Pharmacol..

[44] Cassar S., Beekhuijzen M., Beyer B., Chapin R., Dorau M., Hoberman A., Krupp E., Leconte I., Stedman D., Stethem C. (2019). A multi-institutional study benchmarking the zebrafish developmental assay for prediction of embryotoxic plasma concentrations from rat embryo–fetal development studies. Reprod. Toxicol..

